# Intracultural Variation in the Knowledge of Medicinal Plants in an Urban-Rural Community in the Atlantic Forest from Northeastern Brazil

**DOI:** 10.1155/2012/679373

**Published:** 2011-11-03

**Authors:** Cecília de Fátima Castelo Branco Rangel de Almeida, Marcelo Alves Ramos, Rafael Ricardo Vasconcelos Silva, Joabe Gomes de Melo, Maria Franco Trindade Medeiros, Thiago Antonio de Sousa Araújo, Alyson Luiz Santos de Almeida, Elba Lúcia Cavalcanti de Amorim, Rômulo Romeu da Nóbrega Alves, Ulysses Paulino de Albuquerque

**Affiliations:** ^1^Laboratory of Applied Ethnobotany, Department of Biology, Federal Rural University of Pernambuco, Avenida Dom Manoel de Medeiros s/n, Dois Irmãos, 52171-900 Recife, PE, Brazil; ^2^Natural Products Laboratory, Pharmacy Department, Federal University of Pernambuco, 50670-901 Recife, PE, Brazil; ^3^Ethnozoology, Conservation and Biodiversity Research Group, Departamento de Biologia, Universidade Estadual da Paraíba, 58109-753 Campina Grande, PB, Brazil

## Abstract

This study assessed the intracultural knowledge of the use of medicinal plants in an urban-rural community in an Atlantic forest fragment in northeastern Brazil. We examined the importance of native and exotic species and the effects of gender and age on that knowledge. We also compared data obtained from different groups of informants (local experts and general community). We conducted 194 interviews between June 2007 and January 2008, using the freelist technique and semistructured forms to collect ethnobotanical data. Information obtained from the community was compared with that from six local experts who participated in a survey in 2003. From a total of 209 ethnospecies, exotic and herbaceous plants presented higher richness. With respect to the number of citations, women and older informants were shown to know a higher number of medicinal plants. Comparing knowledge of local experts with that of the general community, we noted that experts know a similar wealth of plant families and therapeutic indications, but the community knows a greater species richness. These results indicate that local experts may provide useful information for studies that search for a quick diagnosis of the knowledge of a given community.

## 1. Introduction

In Brazil, the Atlantic Forest is one of the most biologically diverse ecosystems, responsible for harboring a large number of endemic species [1, 2]. It extends from Rio Grande do Norte to Rio Grande do Sul [3] and, given its location in the coastal area, is currently under strong pressure from real estate speculation. In addition, there are the pressures generated by timber extraction, the cycles of sugar cane, coffee, and gold, and, more recently, the expansion of farming and forestry with exotic species.

Human populations living in the surrounding areas of the Atlantic Forest play an important role in its exploitation as they often rely on forest resources for their subsistence and extract biological resources from it on a daily basis [4]. Understanding how these people use such resources is a task of great current interest, which may contribute to the discovery of products of economic interest and to the conservation of biological resources.

Thus, ethnobotanical studies can contribute to assessing how local knowledge is distributed among members of a community and the relationship between that knowledge and the increase of exotic species in the local repertoire of medicinal plants [5–7].

Common knowledge about plant resources, especially medicinal ones, is highly dynamic and subject to several influences, may vary according to gender, age, education level, income, roles that individuals play within the family, skills, and abilities [8–11], and may represent key elements of the knowledge of the diversity and richness of species [12].

Different social patterns have been reported to impact the knowledge of medicinal plants, emphasizing the need of studies that address such questions. For instance, Almeida et al. [13] did not observe any differences between the knowledge of men and women, whereas age and income were correlated with the number of citations for a given plant and its indication, suggesting that older people with a higher income had greater knowledge about such plant resources.

Thus, the goal of this study was to assess the intracultural knowledge of the use of medicinal plants in an urban-rural community in an Atlantic forest fragment in northeastern Brazil in order to document the importance of native and exotic species within the group of plants mentioned and the effects of gender and age on the knowledge of medicinal plants and to compare the quality of information gathered from different groups of informants (local experts versus general community).

## 2. Materials and Methods

### 2.1. Study Area

The study was conducted at Igarassu, located in the microregion of Itamaracá and the mesoregion of Recife, in Pernambuco state (7°50′20′′ S and 35°00′10′′ W; 20 m a.s.l.), 30 km from the state capital [14–16]. The climate is tropical, hot, and humid, with autumn/winter rains (according to the classification of Köeppen). The average annual temperature is 27°C, and the average annual rainfall is approximately 2,000 mm [14–16]. The municipality has a total area of 304.2 km^2^, with a population of 72,990 people (219.9 inhabitants/km^2^), 74.9% of which live in urban areas [14].

The predominant vegetation is composed of remnants of Atlantic forest, secondary forests, mangroves, palm trees, and areas of commercial and subsistence agriculture. There are ecological reserves in the city, such as the São José Plant Forest, with tall, dense vegetation, located on Transcanavieira Highway (PE-41) and with an area of 323.30 ha [17].

The community studied is known as “Três Ladeiras” and is located on the lands of the “São José Plant,” a sugar refinery. The “São José Plant” is surrounded by Atlantic forest fragments belonging to an ecological reserve [18]. The forest is part of the conservation area of the Botafogo River basin, in accordance with state law no. 9860, which since August 12, 1986, has been aimed at protecting the landscape, soil, and river basin [19]. The fragments occupy a total area of 210 ha [18]. The community lies 30 km north of the county seat and is located at the back of a large hill, whose extension contains three elevations that give the community its name. The district has 1,794 inhabitants, of which 1,077 live in urban areas and 687 in rural ones [20].

Most men from the community work at the plant although the number of people employed by the refinery oscillates during the year, increasing and decreasing according to season and periods of land preparation, planting, and harvesting [15]. It is not unusual to find among the residents of the community families with small fields that provide nutritional and/or economic support during periods when there is no work at the plant [15]. There is no sanitation, medical care takes place in a health clinic for minor health problems, and disease control is provided by health workers through weekly home visits. Patients who require extra care are relocated to hospitals in the county seat of Igarassu.

### 2.2. Data Collection

Ethnobotanical data were obtained through the freelist technique, followed by semistructured interviews [21]. The interviews were conducted with the senior member of the family, over 18 years old, present on the visit of the interviewer. Initially, we obtained a Term of Informed Consent from those willing to participate in the study in accordance with the legal and ethical aspects of Resolution 196/96 from the Ethics and Research Committee [22].

Because the community had a large number of residents, we sampled 51% of all households and conducted 194 interviews (140 women and 54 men) between June 2007 and January 2008. The age of informants ranged from 18 to 93 years. For the interviews, one main question was asked: “What medicinal plants do you know?”. In a second event, we gathered information on each species mentioned, the part of the plant used, the method of preparation, its indication and contraindication, as well as socioeconomic data from informants, such as gender, age, family income, and number of residents in the household. Ages were grouped into five different groups, ranging from 18 to over 68 years.

We used the data obtained in this study and in the work of Gazzaneo et al. [15] to compare the information obtained from the general community and local experts, respectively. The latter study was conducted in the same community in 2003 and was attended by six informants identified as “local experts,” given their more detailed knowledge on the use of medicinal plants [23]. This group of informants was composed of three men and three women, with ages ranging from 51 to 102 years. The data sampling performed by Gazzaneo et al. [15] was intentionally nonrandom and assumed that local experts provide more specific, high-quality information about medicinal plants. To select this group of informants, the authors used the “snowball” method [24]. Data were collected using semistructured interviews that gathered information related to the knowledge of medicinal plants.

### 2.3. Species Categorization and Indications Mentioned by Informants

All plants mentioned during interviews were identified and classified as either native or exotic species according to their biogeographical origin. We considered native species those endemic to the study region and also native to South America. Exotic species were considered to be those of extracontinental origin, cultivated in the region, and widely distributed, such as tropical invasive and cosmopolitan species.

To calculate the relative importance of species, all indications mentioned by the informants were grouped into 18 disease categories, according to the classification from the World Health Organization [25]: digestive, respiratory, gynecological/urinary, circulatory, nervous, sensory, motor, puerperium, cutaneous, scarring, poisoning, neoplasia, hematopoietic, nutritional, infectious/parasitic, sexual inappetence and antiabortion, and postpartum. Diseases not categorized by the aforementioned system were grouped into the category “undefined ailments and pains” by virtue of their symptoms and signs of multiple origins [26].

All species mentioned by informants, excluding those commercialized, were collected, identified, and deposited in the herbaria of Professor Geraldo Mariz (UFP), at the Federal University of Pernambuco, Professor Dárdano de Andrade Lima (IPA), at the Agricultural Research Company, and Professor Sérgio Tavares (HST), at the Federal Rural University of Pernambuco.

### 2.4. Data Analysis

We calculated the value of relative importance (RI) for all species [27] with the following formula: RI = NBS + NP, where NBS is the number of body systems treated by a particular species (NBSS) divided by the total number of body systems treated by the most versatile species (NBSVS) and NP is the number of attributed properties of a particular species (NPS) divided by the total number of properties attributed to the most versatile species (NPVS).

The chi-squared adherence test was used to check for differences between the following factors: number of native versus exotic plants and number of plants observed in each life form (herb, shrub, and tree). We also compared the richness of exclusive species between different age groups, richness of families, total number of mentioned species, and number of exclusive species between local experts and the general community.

We used the Kruskal-Wallis nonparametric test to test for differences in the richness of ethnospecies and mentioned indications between men and women and between each age group and to check for differences between the relative importance of species mentioned by local experts and the general community.

We used Williams' *G*-test to compare the proportion of the number of native and exotic species (exclusive or not) mentioned by local experts and the general community.

The Spearman correlation test was applied to check for the relationship between the number of ethnospecies and the number of mentioned indications according to the age of the informants and to check for a relationship between relative importance (RI) of species mentioned by the general community and the RI calculated for local experts.

All statistical analyses were performed using the statistical package BioEstat 5.0 [28].

## 3. Results

### 3.1. Richness of Medicinal Plants Mentioned by Informants

In total, 209 ethno-species were mentioned during interviews; 151 were identified to the species level and 21 to the genus level only (Table 1). The plants were distributed in 74 families, and most families (66%) were represented by up to two species. The most represented families were Lamiaceae (10 spp.); Caesalpiniaceae and Curcubitaceae (8 spp.); Asteraceae, Euphorbiaceae, and Mimosaceae (5 spp.).

**Figure 1:**
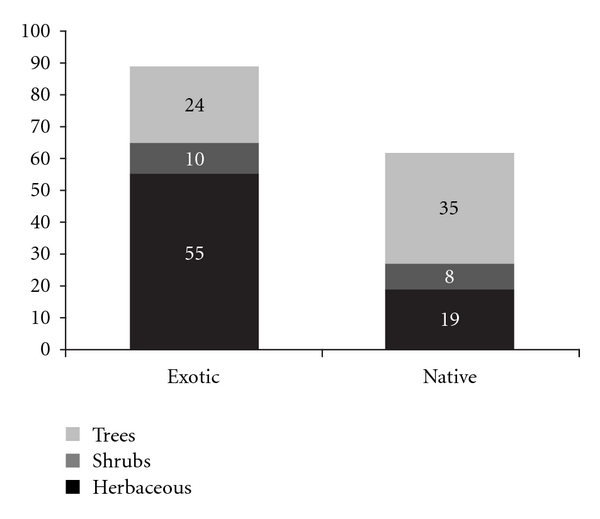
Origin and life form of the medicinal species mentioned in the community of Três Ladeiras, Igarassu, Pernambuco state, northeastern Brazil.

With respect to the origin of the identified species, we observed that 89 were exotic and 62 were native (Figure 1), and the difference was statistically significant (*χ* ^2^ = 4.8; *P* < 0.05). That result indicates that informants knew more exotic plants that could be used for medicinal purposes. With respect to the life form of plants, there was a predominance of herbs (74), followed by trees (59) and shrubs (18) (Figure 1), but we only observed statistical differences when we compared the richness of shrubs with that of herbs (*χ* ^2^ = 34.01; *P* < 0.0001) and trees (*χ* ^2^ = 21.83; *P* < 0.0001). The number of herbs and trees was not significantly different (*χ* ^2^ = 1.7; *P* = 0.23), indicating that the richness of herbs and trees was similar in the pool of plants mentioned by informants. However, when considering the distribution of species according to their origin, we observed a different pattern: for exotic plants, there was a higher number of herbaceous plants compared to the other two life forms (shrubs: *χ* ^2^ = 31.15, *P* < 0.0001; trees: *χ* ^2^ = 12.16, *P* = 0.005), whereas, for native plants, there was a higher number of trees (herbs: *χ* ^2^ = 4.7, *P* = 0.02; shrubs: *χ* ^2^ = 16.95, *P* < 0.0001).

The most mentioned species were *Schinus terebinthifolius* Raddi (aroeira-219 citations), *Alpinia zerumbet* (Pers.) B. L. Burtt & R. M. Sm. (colônia-190 citations), *Pithecellobium cochliocarpum* (Gomez) Macbr. (babatenon-183), *Plectranthus amboinicus* (Lour.) Spreng (hortelã graúda-155), *Mentha piperita* L. (hortelã miúda-141), and *Cymbopogon citratus* (DC.) Stapf (capim santo-133) (Table 1). Except for *S. terebinthifolius* and *P. cochliocarpum*, all these plants are exotic, emphasizing the importance of exotic plants to the knowledge of medicinal plants in the region.

### 3.2. Influence of Gender and Age on the Knowledge of Medicinal Plants

There were significant differences in the knowledge of medicinal plants according to gender, with women knowing a higher richness of ethno-species (*H* = 117.29; *P* = 0.0006) and indications (*H* = 134; *P* = 0.0003). They mentioned a total of 166 ethno-species, with a mean number of 13.33 ± 7.84 citations per person, and a total of 146 indications, with a mean of 10.16 ± 5.73 citations per person, whereas men mentioned 136 ethno-species and 93 indications, with a mean of 9.95 ± 7.05 and 7.51 ± 4.9 citations per person, respectively.

The number of plants and indications mentioned by each informant correlated with their age (rs = 0.33, *P* < 0.0001; rs = 0.37, *P* < 0.0001, resp.). However, when we compared the average number of ethnospecies and indications in each age group, we observed different patterns (Table 2). The number of known plants only varied in informants from the 18–28-year-old age group, suggesting that the richness of known plants was smaller in younger participants, which may reflect the limited experience and contact of young informants with plant resources from the region. Although informants aged 49–58 years old had greater knowledge of medicinal plants in the region, they were only significantly different from younger informants (Table 2). With respect to the number of indications mentioned in each age group, we observed a similar pattern to the previous one, with younger informants (18–28 yrs.) knowing a smaller variety of indications. However, the knowledge of informants was significantly higher for the age groups 49–58 years old and older than in other age groups.

When we analyzed the influence of gender and age on the distribution of knowledge, we observed a few patterns that often differed from the data presented by the general community. Younger women (18–28 yrs.) also had less knowledge of the richness of medicinal species, while, in other age groups, knowledge was homogeneous. For women, we observed the formation of two groups regarding the number of indications: one group consisting of the three younger groups, with a lower number of indications, and the other consisting of older age groups, with a higher number of indications.

For men, knowledge of plant richness and indications showed a different pattern. The knowledge of informants in the 18–48-year-old age groups did not present any statistical differences. That difference only appeared in the age groups 49–58 years old and older, indicating an increase in the number of species known and the variety of indications occurring only in older age groups, whereas for women it was also observed in younger age groups.

**Figure 2:**
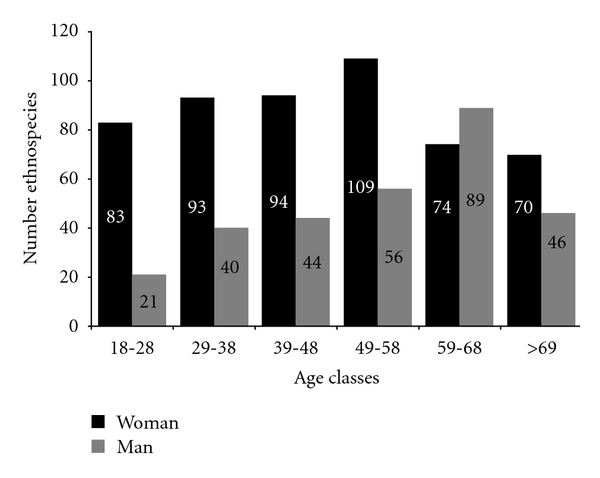
Distribution of the knowledge of medicinal plants between men and women in the community of Três Ladeiras, Igarassu, Pernambuco state, northeastern Brazil.

We observed a continuous increase in the number of ethno-species mentioned with an increase in age, for both men and women, when total plant richness was considered (Figure 2), up to the age group with the highest richness of plants mentioned. In subsequent age groups, plant richness started to decrease. Among women, the 49–58-year-old age group had the greatest knowledge of plants (109 ethno-species), whereas for men the greatest knowledge of plants was observed in an older class (59–68 yrs.). These results indicate that, in the community of Three Hills, the commitment of women to family care compels them to know, from an early age, a large number of plants with medicinal purposes.

Following the age groups with a higher number of plants, there was a decrease in plant richness in older groups, possibly related to memory loss, which is common among older people. We did not observe the same pattern when the mean number of plants mentioned by informants was analyzed, as previously noted (Table 2). In this case, people still mentioned a high number of citations, even in the oldest age group (>69 yrs.).

Exclusive species were mentioned in all age groups (Table 2), with a total richness of 78 exclusive species distributed in the six age groups. The 49–58- and 59–68-year-old age groups stand out for their higher richness, although there were no statistical differences in the number of exclusive species between age groups (*χ* ^2^ = 9.38; *P* = 0.09), indicating that, in every age group, informants had a repertoire of exclusive plants that was not shared by people from other age groups.

### 3.3. Analysis of the Knowledge of Medicinal Plants between Local Experts and the General Community

**Figure 3:**
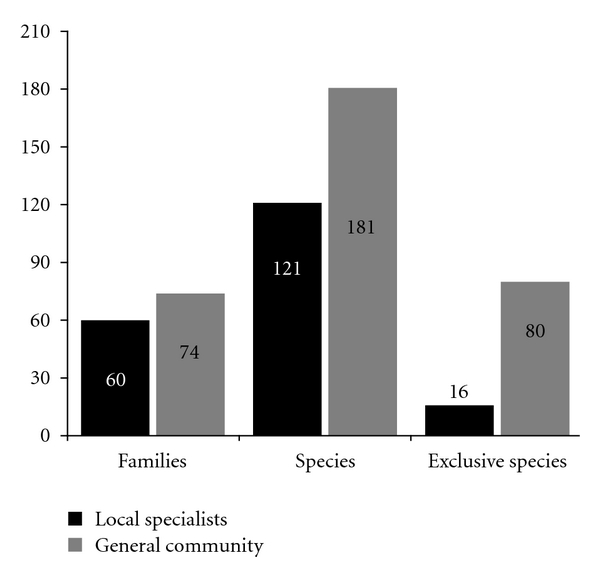
Comparison of the richness of families, species, and exclusive species between local experts and the general community.

Data obtained from the general community presented a higher richness of plant families and medicinal species when compared with data obtained from local experts. The difference was highly significant for total species richness (*χ* ^2^ = 11.921, *P* = 0.0006) and exclusive species richness (*χ* ^2^ = 42.667, *P* = 0.0001) but was not significantly different for the richness of plant families (*χ* ^2^ = 1.463, *P* = 0.2265, Figure 3). The results indicate that the knowledge of local experts managed to represent the richness of useful plant families cited by the general community but not the total number of species (Table 3). However, the most species (84.2%) mentioned by at least 20 informants from the general community were also mentioned by local experts. This result indicates that expert informants mentioned medicinal plant species that are better known among other members of the community. 

The numbers of exotic and native species mentioned by local experts and that by the general community were not significantly different according to Williams' *G*-test (*G* = 0.9369, *P* = 0.3331). Among exclusive species, we also did not observe any significant differences between native and exotic plants (*G* = 0.153, *P* = 0.6957), suggesting that local experts and the general community presented a similar citation repertoire of native and exotic species.

There was no significant difference between the relative importance (RI) of species mentioned by local experts and that by general community (*H* = 0.7899, *P* = 0.3741). We observed a significant correlation between local experts and the general community in the number of indications per species according to the Spearman correlation test (rs = 0.515, *P* < 0.0001). This result suggests that local experts and the general community provided similar information regarding the indication of medicinal plant species. Among the ten species with higher RI mentioned by local experts and the general community, the species *Borreria verticillata* L. G. Mey., *Hymenaea martiana* Hayne, *Mentha piperita* L., *Pithecellobium cochliocarpum* (Gomez) Macbr., and *Schinus terebinthifolius* Raddi occurred in both studies. These results show that using local experts to provide information on the indications of medicinal plants was useful in the given context. However, the same was not observed for data on species richness. Thus, we recommend engaging the whole community to gather such data. Such precaution may prevent a large number of known species from being neglected, as in our study.

## 4. Discussion

### 4.1. Richness of Medicinal Plants Mentioned by Informants

The study showed high diversity in the knowledge of medicinal plants, with significant results for species prescribed for basic health care. The acceptance of folk medicine and the limited access to public healthcare services in the community may be factors contributing to the knowledge of medicinal species in local medical practices.

A few plant families prominent in this study, such as Lamiaceae and Asteraceae, are reported as very diverse taxonomic groups in the literature. Their high diversity probably reflects a greater amount of bioactive compounds [29], which may explain their prominence in this study and in similar ones in other regions [30, 31]. 

Most medicinal plants used in the study area are exotic herbs that usually grow in anthropogenic areas, such as agricultural fields, gardens, and roads. Other studies performed in forest environments in different regions have shown that traditional communities select anthropogenic areas as important resource sources [32–36]. The frequent citation of herbs in the community of “Três Ladeiras” may be a consequence of the importance of this life form in anthropogenic areas, also due to the presence of strong bioactive compounds. In a study conducted in the same community, Gazzaneo et al. [15] also reported that more of the medicinal plants used by experts were herbs, collected mainly in the backyards of homes and small farms.

According to Voeks [37], weeds are often abundant in easily accessible places and rich in bioactive compounds, and as a result they are widely represented in tropical medicinal floras.

### 4.2. Influence of Gender and Age on the Knowledge of Medicinal Plants

Women had greater knowledge of medicinal plants when compared to men in the community studied. That result was probably due to women being the caregivers for their families, a trend also observed in other studies [5, 38–40]. This scenario may also reflect the different activities performed by men and women in the community because the latter must dedicate themselves to their homes and families, which help them assimilate the knowledge they will need to keep their homes healthy at an earlier age. We should add that most species from the list of mentioned plants were exotic and herbaceous plants that are found in places women are more familiar with, such as backyards.

The study showed that young informants had less knowledge of medicinal plants when compared to older ones, which can be attributed to a lack of interest in learning and practicing such knowledge in younger generations, who are increasingly influenced by modernization. Several studies have reported similar results [38, 41–45]. It is also important to notice that older people are more experienced and have had greater contact with plant resources and time to exchange knowledge with other informants from the region. Moreover, older people are more often affected often by various illnesses, which may help to increase their repertoire of plants and indications. In addition, they are responsible for preparing home remedies for themselves and for younger people, favoring the retention of knowledge and prompting younger individuals to use the plant resource without necessarily having knowledge of the remedy or its preparation.

### 4.3. Analysis of the Knowledge of Medicinal Plants between Local Experts and the General Community

This research showed that studies focused on experts can generate useful information, with a satisfactory level of reliability. But these are eminently suitable for quick diagnosis about the knowledge and use of medicinal plants in a community. That approach has the advantage of minimizing costs and time when collecting ethnobotanical data in the community surveyed. However, studies aiming to gather such information in greater detail should ideally engage other members of the community. That may prevent a large number of known species from being neglected. Vandebroek [46] reports that the careful selection of informants is a key task of the ethnobiologist and cannot be a simple step. The author suggests that, for a scientifically rigorous research, should be involved as many participants as possible, but if time is really a limiting factor, it is necessary to select key informants who have a high degree of knowledge about plants in the region as well as a high level of consensus with others.

Within this scenario, the association between both groups of informants is also possible [45–47]. In fact, the information gathered from key informants (experts) in those studies helped prepare semistructured forms and consensus analyses among informants. In some cases, local experts are used as facilitators for data collection, accompanying the researcher during interviews with other members of the community. These studies serve as examples of the advantages and limitations of different informant profiles and emphasize the importance of clearly establishing the goals the researcher hopes to achieve to best engage the most appropriate informants.

It should be noted, additionally, that there was a five-year interval between the data obtained from local experts and the general community. The influence of such a gap could not be measured or controlled, given the sampling design of each study and the dynamic character of human knowledge, and this limitation restricts the possibility of extrapolating the considerations discussed here. Moreover, we could not completely rule out the possibility that the so-called local expert informants have been added to the sample of the present study.

## Figures and Tables

**Table 1:** Medicinal plants mentioned in the community of Três Ladeiras, Igarassu, Pernambuco state, Brazil.

Family/scientific name	Vernacular name	Habit	Origin	Citation number	RI 2005*	RI 2008
Acanthaceae						
*Justicia pectoralis *Jacq.	Chambá	Herbs	Native	21	0.33	0.37
*Justicia *sp.	Anador	—	—	25	—	1.00
*Graptophyllum pictum *(L.) Griff.	Melacilina	Herbs	Exotic	15	0.67	0.94

Amaranthaceae						
*Alternanthera brasiliana *(L.) Kuntze.	Novalgina	Herbs	Exotic	3	—	0.35
*Pfaffia glomerata *(Spreng.) Pederson	Acônico/Acônito	Herbs	Exotic	17	0.67	0.27

Anacardiaceae						
*Anacardium occidentale *L.	Cajueiro roxo	Tree	Native	113	0.33	1.24
*Mangifera indica *L.	Manga	Tree	Exotic	11	0.83	0.62
*Schinus terebinthifolius *Raddi	Aroeira	Tree	Native	219	1.17	1.60
*Spondias purpurea *L.	Siriguela	Tree	Native	3	—	0.27

Annonaceae						
*Annona montana *Macfad.	Aticum	Tree	Native	13	0.83	0.61
*Annona muricata *L.	Graviola	Tree	Exotic	4	—	0.30
*Annona squamosa *L.	Pinha	Tree	Exotic	1	—	0.12

Apiaceae						
*Daucus carota *L.	Cenoura	Herbs	Exotic	1	—	0.12
*Foeniculum vulgare *Mill.	Endro	Herbs	Exotic	12	—	0.74
*Pimpinella anisum *L.	Erva doce	Herbs	Exotic	45	0.33	1.09

Apocynaceae						
*Catharanthus roseus* L. G. Don.	Boa noite branca	Herbs	Exotic	3	—	0.27
*Hancornia speciosa *Gomes	Mangaba	Tree	Native	1	0.17	0.12

Arecaceae						
*Acrocomia aculeata *(Jacq.) Lodd. ex Mart.	Macaiba	Tree	Native	30	0.17	0.76
*Cocos nucifera *L.	Coco amarelo	Tree	Exotic	7	0.17	0.39
*Elaeis guineensis *Jacq.	Dendezeiro	Tree	Exotic	2	0.17	0.24
*Syagrus *sp.	Coco catolé	Tree	Native	6	0.50	0.42

Asteraceae						
*Acanthospermum hispidum *DC.	Espinho de cigano	Herbs	Exotic	48	0.67	0.76
*Conyza bonariensis* (L.) Cronq.	Rabo de raposa	Herbs	Exotic	3	0.33	0.12
*Helianthus annuus *L.	Girassol	Herbs	Native	10	0.17	0.12
*Matricaria chamomilla *L.	Camomila	Herbs	Exotic	7	—	0.12
*Pluchea *sp.	Mar de cravo	Herbs	—	1	—	0.12
*Gymnanthemum amygdalinum *(Delile) Sch.Bip. ex Walp.	Alcachofra	Herbs	Exotic	50	0.33	0.83
Asteraceae 1	Carqueja	—	—	2	—	0.12

Begoniaceae						
*Begonia reniformis *Dryand.	Capeba	Shrub	Native	3	0.17	0.24

Bignoniaceae						
*Handroanthus impetiginosus *(Mart. ex DC.) Mattos	Pau daico roxo	Tree	Native	8	0.67	0.54

Bombacaceae						
*Chorisia *sp.	Barriguda	Tree	Native	1	—	0.12

Boraginaceae						
*Heliotropium angiospermum *Murray	Crista de galo	Herbs	Exotic	1	—	0.12
*Heliotropium indicum* L.	Fedegoso	Herbs	Exotic	3	0.50	0.35

Brassicaceae						
*Nasturtium officinale *R. Br.	Agrião	Herbs	Exotic	48	0.83	0.53

Bromeliaceae						
*Ananas comosus *(L.) Merr.	Abacaxi	Herbs	Native	14	—	0.39
*Tillandsia usneoides *(L.) L.	Salambaia	Herbs	Native	2	0.17	0.24

Burseraceae						
*Protium heptaphyllum *(Aubl.) Marchand	Amescla	Tree	Native	1	1.00	0.12

Cactaceae						
*Cereus jamacaru *DC.	Cardeiro	Tree	Native	5	—	0.12

Caesalpiniaceae						
*Bauhinia forficata *Link	Pata de vaca	Tree	Native	9	—	0.30
*Caesalpinia echinata *Lam.	Pau brasil	Tree	Native	1	—	0.12
*Caesalpinia ferrea *Mart. ex Tul.	Jucá	Tree	Native	11	0.50	0.27
*Copaifera* sp.	Pau dóleo	Tree	—	1	0.17	0.12
*Hymenaea martiana *Hayne	Jatobá	Tree	Native	27	1.33	1.20
*Senna alata *(L.) Roxb.	Café beirão	Shrub	Exotic	1	—	0.12
*Senna occidentalis *(L.) Link.	Manjirioba/Mata pasto	Herbs	Exotic	24	0.17	0.76
*Poincianella pyramidalis *(Tul.) L. P. Queiroz	Catingueira	Tree	Native	1	—	0.12

Capparaceae						
*Cleome spinosa *Jacq.	Mussambê	Shrub	Exotic	11	0.17	0.30

Caprifoliaceae						
*Sambucus nigra *L.	Flor de sabugo/Sabugueiro	Tree	Exotic	33	0.50	0.57

Caricaceae						
*Carica papaya *L.	Mamão	Tree	Native	7	0.33	0.46

Caryophyllaceae						
*Dianthus caryophyllus *L.	Cravo branco	Herbs	Exotic	4	—	0.27

Cecropiaceae						
*Cecropia palmata *Willd.	Embauba branca	Tree	Native	6	0.33	0.42

Chenopodiaceae						
*Beta vulgaris *L.	Beterraba	Herbs	Exotic	8	—	0.34
*Chenopodium ambrosioides *L.	Mastruz/Mentruz	Herbs	Exotic	85	0.67	0.68

Chrysobalanaceae						
*Licania *sp.	Oiti/Oiticica	—	—	2	—	0.24
Chrysobalanaceae 1	Oiticoró	—	—	1	—	0.12

Clusiaceae						
*Vismia guianensis *(Aubl.) Pers.	Lacre	Tree	Native	12	0.33	0.42

Combretaceae						
*Buchenavia* sp.	Imbiriba	—	—	4	0.33	0.15
*Terminalia catappa *L.	Coração de negro	Tree	Exotic	2	—	0.58

Convolvulaceae						
*Ipomoea asarifolia *(Ders.) R. et Sch	Salsa	Herbs	Exotic	1	0.17	0.15
*Operculina alata *(Ham.) Urb.	Batata de purga	Herbs	Native	1	0.50	0.12
Convolvulaceae 1	Acanfó/Acafú	—	—	10	0.33	0.34
Convolvulaceae 2	Sassá	—	—	1	—	0.12

Crassulaceae						
*Kalanchoe laciniata *(L.) DC.	Corona branca	Herbs	Exotic	17	—	0.81
*Kalanchoe *sp.	Corona roxa	—	—	6	—	0.51

Cucurbitaceae						
*Citrullus vulgaris *Schard.	Melância	Herbs	Exotic	1	—	0.12
*Cucumis anguria *L.	Maxixe	Herbs	Exotic	1	—	0.12
*Cucumis melo *L.	Melão	Herbs	Exotic	1	—	0.12
*Cucumis sativus *L.	Pepino	Herbs	Exotic	2	—	0.24
*Curcubita pepo *L.	Jerimum	Herbs	Exotic	8	—	0.24
*Luffa operculata *L. Cong.	Cabacinha	Herbs	Native	1	0.17	0.12
*Momordica charantia *L.	Melão de são caetano	Herbs	Exotic	1	—	0.12
*Sechium edule *(Jacq.) Sw.	Chuchu	Herbs	Exotic	20	0.17	0.27

Equisetaceae						
*Equisetum *sp.	Cavalinha	Herbs	—	1	—	0.12

Euphorbiaceae						
*Cnidosculus urens *(L.) Arthur	Urtiga branca	Herbs	Native	25	0.50	0.68
*Euphorbia tirucalli *L.	Aveloz	Shrub	Exotic	1	—	0.12
*Jatropha gossypiifolia *L.	Pinhão roxo	Shrub	Exotic	5	—	0.35
*Jatropha mollissima *(Pohl.) Baill.	Pinhão branco	Shrub	Native	2	—	0.24
*Manihot esculenta *Crantz	Macacheira/Roça	Herbs	Native	1	—	0.12
*Phyllanthus niruri *L.	Quebra pedra	Herbs	Exotic	13	0.17	0.46
*Ricinus communis *L.	Carrapateira/Mamona	Shrub	Exotic	5	—	0.27

Fabaceae						
*Bowdichia virgilioides* Kunth	Sucupira	Tree	Native	5	0.50	0.39
*Vicia faba *L.	Fava	Herbs	Exotic	1	—	0.12
*Zornia diphylla *(L.) Pers.	Urinana	Herbs	Native	3	0.17	0.15

Flacourtiaceae						
Flacourtiaceae 1	Imbira branca	—	—	2	—	0.12

Heliconiaceae						
*Heliconia psittacorum *L. f.	Paquivira	Herbs	Native	1	—	0.12

Illiaceae						
*Illicium verum *Hook. f.	Anil estrelado	Tree	Exotic	9	—	0.73

Iridaceae						
*Crocus *sp.	Açafrão	Herbs	—	1	0.33	0.12
*Eleutherine bulbosa *(Mill.) Urb.	Alho do mato	Herbs	Native	3	0.67	0.15

Lamiaceae						
*Aeollanthus suaveolens *Mart. ex Spreng.	Macassá	Herbs	Exotic	32	1.33	1.13
*Mentha piperita *L.	Hortelã miúda	Herbs	Exotic	141	0.83	2.00
*Mentha pulegium *L.	Hortelã pastilha/H. vick	Herbs	Exotic	34	0.17	1.07
*Ocimum basilicum *L.	Manjericão/Manjericão são josé	Herbs	Exotic	50	0.50	1.16
*Ocimum basilicum* var. *minimum *(Willd.) Benth.	Manjericão miúdo	Herbs	Exotic	5	0.17	0.35
*Ocimum campechianum *Mill.	Alfavaca de caboclo	Herbs	Native	14	—	0.69
*Ocimum gratissimum *L.	Louro falso/L. caseiro/ Hortelã fernando/H. são severino	Herbs	Exotic	4	0.17	0.24
*Plectranthus amboinicus *(Lour.) Spreng.	Hortelã graúda/H. gorda/H. bahia	Herbs	Exotic	155	0.83	1.22
*Plectranthus barbatus *Andrews	Boldo caseiro/Boldo falso/Hortelã caboclo	Herbs	Exotic	17	0.50	0.84
*Rosmarinus officinalis *L.	Alecrim	Herbs	Exotic	11	0.17	0.96
Lamiaceae 1	Alfazema	Herbs	—	1	—	0.12
Lamiaceae 2	Alfazema de caboclo	Herbs	—	1	—	0.12
Lamiaceae 3	Veiga morta	Herbs	—	53	0.50	0.84

Lauraceae						
*Nectandra cuspidata *Ness & Mart.	Canela	Tree	Native	71	—	0.95
*Persea americana *Mill.	Abacate	Tree	Exotic	15	0.83	0.62

Liliaceae						
*Allium cepa *L.	Cebola	Herbs	Exotic	12	0.67	0.34
*Allium sativum *L.	Alho	Herbs	Exotic	19	0.33	0.56
*Aloe vera *(L.) Berm.f.	Baborsa/Erva babosa	Herbs	Exotic	32	0.83	1.13

Loranthaceae						
Loranthaceae 1	Cipó estanca sangue	—	—	1	0.33	0.12

Malpighiaceae						
*Byrsonima sericea *DC.	Murici	Tree	Native	1	—	0.12
*Malpighia glabra *L.	Acerola	Tree	Exotic	10	—	0.51

Malvaceae						
*Gossypium barbadense *L.	Algodão	Shrub	Exotic	2	0.50	0.20
*Uerna lobata *L.	Malva rosa	Herbs	Native	11	0.33	0.51

Meliaceae						
*Cedrela odorata *L.	Cedro	Tree	Native	1	—	0.12

Mimosaceae						
*Acacia *sp.	Espinheiro	Tree	—	3	—	0.12
*Anadenanthera colubrina *(Vell.) Brenan.	Angico	Tree	Native	3	—	0.24
*Inga bahiensis *Benth.	Inga porco	Tree	Native	1	—	0.12
*Mimosa tenuiflora* (Willd.) Poir.	Jurema preta	Tree	Native	2	—	0.12
*Piptadenia stipulacea *(Benth.) Ducke.	Jurema branca	Tree	Native	1	—	0.12
*Pithecellobium cochliocarpum *(Gomez) Macbr.	Babatenom	Tree	Native	183	1.83	1.14
*Pithecellobium saman* var. *acutifolium *Benth.	Budão de velho	Tree	Native	2	—	0.24

Monimiaceae						
*Peumus boldus *Mol.	Boldo do chile	Herbs	Exotic	44	—	0.73

Moraceae						
*Artocarpus communis *J.R. Forst. & G. Forst.	Fruta pão	Tree	Exotic	3	—	0.35
*Artocarpus integrifolia *L. f.	Jaca	Tree	Exotic	2	—	0.24
*Dorstenia *sp.	Conta erva	—	—	1	0.83	0.12

Musaceae						
*Musa paradisiaca *L.	Bananeira	Tree	Exotic	19	0.33	0.73

Myrtaceae						
*Eucalyptus citriodora *Hook.	Eucalipto	Tree	Exotic	7	0.17	0.24
*Eugenia uniflora *L.	Pitanga	Tree	Native	58	0.17	0.59
*Psidium guajava *L.	Goiaba	Tree	Native	51	0.17	0.24
*Psidium guineense *Sw.	Araça	Shrub	Native	5	0.17	0.12
*Syzygium aromaticum *(L.) Merr. & L.M. Perry	Cravo do reino	Tree	Exotic	5	—	0.59
*Syrygium jambolanum *(Lam.) DC.	Azeitona preta	Tree	Exotic	14	0.17	0.69

Nyctaginaceae						
*Boerhavia diffusa *L.	Pega pinto	Herbs	Exotic	22	1.17	0.83
*Guapira *sp.	João mole	Tree	—	3	—	0.24

Olacaceae						
*Ximenia americana *L.	Ameixa	Tree	Native	1	0.17	0.12

Oxalidaceae						
*Averrhoa carambola *L.	Carambola	Tree	Exotic	17	0.83	0.30

Papaveraceae						
*Argemone mexicana *L.	Cardo santo	Herbs	Exotic	7	0.50	0.24

Passifloraceae						
*Passiflora edulis *Sims.	Maracujá	Shrub	Native	12	0.17	0.49

Pedaliaceae						
*Sesamum orientale *L.	Gergelim preto/Gigilim	Herbs	Exotic	4	0.17	0.24

Phytolacaceae						
*Petiveria alliacea *L.	Timpi	Herbs	Native	7	0.67	0.54

Piperaceae						
*Peperomia pellucida *H.B.K.	Lingua de sapo	Herbs	Native	5	—	0.39
*Piper nigrum *L.	Pimenta do reino	Shrub	Exotic	2	—	0.24

Poaceae						
*Cymbopogon citratus *(DC.) Stapf	Capim santo	Herbs	Exotic	133	0.50	1.47
*Imperata brasiliensis *Trin.	Sapé	Herbs	Native	5	—	0.15
*Phalaris canariensis *L.	Alpiste	Herbs	Exotic	4	0.17	0.24
*Zea mays *L.	Milho	Herbs	Exotic	2	—	0.12

Polygalaceae						
*Polygala *sp.	Esquentado	—	—	1	—	0.12

Punicaceae						
*Punica granatum *L.	Romã	Tree	Exotic	14	—	0.49

Rhamnaceae						
*Zizyphus joazeiro *Mart.	Juá	Tree	Native	22	0.83	0.76

Rosaceae						
*Pyrus malus *L.	Maçã	Tree	Exotic	1	—	0.12
*Rosa *sp.1	Rosa amélia	—	—	1	—	0.12
*Rosa* sp.2	Rosa branca	—	—	7	0.33	0.62

Rubiaceae						
*Borreria verticillata *L. G. Mey.	Vassoura de botão	Herbs	Exotic	47	1.17	1.51
*Genipa americana *L.	Jenipapo	Tree	Native	53	—	0.64
*Uncaria tomentosa *(Willd. ex Roem. & Schult.) DC.	Unha de gato	Tree	Native	1	—	0.12

Rutaceae						
*Citrus limetta *Risso	Lima	Tree	Exotic	1	—	0.12
*Citrus limonia *Osbeck	Limão	Tree	Exotic	14	—	0.57
*Citrus sinensis *(L.) Osbeck	Laranja	Tree	Exotic	44	0.83	0.93
*Ruta graveolens *L.	Arruda	Herbs	Exotic	75	0.83	1.08

Sapindaceae						
*Cardiospermum halicacabum *L.	Cipó de vaqueiro	Shrub	Native	10	0.33	0.24
*Cupania *sp.	Cabotam	—	—	1	0.33	0.12
*Serjania *sp.	Cipó cururu	—	—	2	—	0.12

Sapotaceae						
*Achras zapota *L.	Sapoti	Tree	Exotic	1	—	0.12
*Sideroxylon obtusifolium *(Roem. & Schult.) T.D. Penn.	Quixaba	Tree	Native	27	—	1.14

Smilacaceae						
*Smilax rotundifolia *L.	Cipó japecanga	Herbs	Native	6	0.17	0.12

Solanaceae						
*Capsicum frutescens *L.	Pimenta	Shrub	Native	3	—	0.24
*Nicotiana tabacum *L.	Fumo	Herbs	Native	1	—	0.12
*Solanum americanum *Mill.	Avamoura/Erva moura	Herbs	Exotic	5	0.17	0.30
*Solanum paniculatum *L.	Jurubeba	Shrub	Exotic	4	1.00	0.35

Sterculiaceae						
*Guazuma ulmifolia *L.	Mutamba	Tree	Native	7	—	0.24

Theacaea						
*Camellia sinensis *(L.) Kuntze	Chá preto	Shrub	Exotic	4	—	0.20

Turneracea						
*Turnera ulmifolia *L.	Xanana	Herbs	Exotic	1	0.17	0.12

Verbenaceae						
*Lantana camara *L.	Chumbinho	Shrub	Native	5	0.33	0.19
*Lippia alba *(Mill.) N.E.Br.	Cidreira/Erva cidreira	Herbs	Exotic	94	0.67	1.86
*Stachytarpheta elatior *Schrad. ex Schult	Mocotó	Herbs	Native	4	0.67	0.12
*Vitex agnus-castus *L.	Liamba	Tree	Exotic	2	0.17	0.24

Violaceae						
*Hybanthus sp.*	Pepaconha	—	—	7	—	0.51

Vitaceae						
*Cissus verticillata *(L.) Nicolson & C.E. Jarvis	Insulina	Shrub	Native	5	0.17	0.12
*Leea *sp.	Café	—	—	3	—	0.27
*Vitis vinifera *L.	Uva	Shrub	Exotic	1	—	0.12

Zingiberaceae						
*Alpinia zerumbet *(Pers.) Burt. ex R. M. Smith	Colônia/Colonha	Herbs	Exotic	190	1.67	0.91
*Costus *sp.	Cana de macaco	—	—	27	—	0.84
*Zingiber officinalis *Rosc.	Gengibre vermelho	Herbs	Exotic	1	—	0.12

Unidentified						
Unidentified 1	Abre caminho	—	—	2	—	0.12
Unidentified 2	Açafroa	—	—	1	—	
Unidentified 3	Boca torta	—	—	1	—	0.12
Unidentified 4	Bugre	—	—	2	—	0.12
Unidentified 5	Cafofa	—	—	1	—	0.12
Unidentified 6	Canela de viado	—	—	1	—	0.12
Unidentified 7	Chumbinho branco	—	—	1	—	0.12
Unidentified 8	Cipó de boi	—	—	2	—	0.24
Unidentified 9	Imbira vermelha	—	—	2	—	0.12
Unidentified 10	Malicia branca	—	—	2	—	0.24
Unidentified 11	Malicia boi/M. fina	—	—	4	—	0.15
Unidentified 12	Malva ferro	—	—	1	—	0.12
Unidentified 13	Moça	—	—	1	—	0.12
Unidentified 14	Pé de galinha/Papo de peru	—	—	1	—	0.12
Unidentified 15	Pega rapaz	—	—	1	—	0.12
Unidentified 16	Perpetua branca	—	—	1	—	0.12
Unidentified 17	Piripiri	—	—	1	—	0.12
Unidentified 18	Piriquiti	—	—	1	—	0.12
Unidentified 19	Quebra faca	—	—	5	—	0.27
Unidentified 20	Quentão	—	—	1	—	0.12
Unidentified 21	Rasteira	—	—	1	—	0.12
Unidentified 22	Rasteirinho	—	—	1	—	0.12
Unidentified 23	Salsa caroba	—	—	1	—	0.12
Unidentified 24	Sete casco	—	—	1	—	0.12
Unidentified 25	Tatajuba	—	—	1	—	0.12

*(—) denotes the absence of the species on the list of Gazzaneo et al. [15].

RI 2005: relative importance calculated from information given by local experts; RI 2008: relative Importance calculated from information given by the general community.

**Table 2:** Distribution of the knowledge of medicinal plants by age group and gender in the community of Três Ladeiras, Igarassu, Pernambuco state, northeastern Brazil.

Age groups	NI	General average number of ethno-species	General average number of indications	NMI	NFI	Average number of cited ethno-species		Average number of cited indications		Total diversity of cited species	Number of exclusive species
						Male	Female	Male	Female		
		*χ* ± SD*	*χ* ± SD*			*χ* ± SD*	*χ* ± SD*	*χ* ± SD*	*χ* ± SD*		
18–28	43	8.8 ± 6^a^	6.3 ± 3.6^a^	8	35	5.37 ± 1.9^a^	9.57 ± 6.4^a^	4 ± 1.2^a^	6.83 ± 3.8^a^	85	10
29–38	44	11.66 ± 6.2^b^	8.84 ± 4.6^b^	8	36	7.6 ± 3.7^ac^	12.55 ± 6.3^b^	5.75 ± 2^ac^	9.53 ± 4.8^b^	103	11
39–48	41	11.98 ± 6.6^b^	9 ± 4.9^b^	12	29	8.83 ± 4.3^ad^	13.28 ± 7^b^	7.67 ± 4.5^ac^	9.66 ± 5.1^b^	99	12
49–58	28	16.39 ± 11^b^	12.79 ± 7.7^c^	7	21	11.71 ± 9.6^bcd^	17.95 ± 11.3^b^	9.14 ± 6.9^bc^	14 ± 7.8^c^	120	19
59–68	24	14.58 ± 8.8^b^	10.7 ± 6.1^c^	14	10	12.93 ± 9.4^bcd^	16.9 ± 7.8^b^	9.14 ± 6.3^bc^	12.9 ± 5.2^bcd^	112	19
>69	14	14.71 ± 6.7^b^	11.5 ± 4.8^c^	5	9	13.4 ± 7.4^bcd^	15.44 ± 6.7^b^	9 ± 4.5^bc^	12.9 ± 4.6^cd^	87	7

*Equal letters in the same column indicate a lack of statistical differences using the Kruskal-Wallis test (*P* < 0.05). NI: number of informants; NMI: number of male informants; NFI: number of female informants.

**Table 3:** Exclusive species list from the work of Gazzaneo et al. [15].

Family/scientific name	Vernacular name	Habit	Origin	RI
Asteraceae				
*Egletes viscosa *(L.) Less.	Macela	Herbs	Exotic	0.17
*Tagetes *sp.	Cravo de defunto	Herbs	—	0.17

Caesalpiniaceae				
*Senna obtusifolia *(L.) H.S. Irwin & Barnbey	Mata pasto	Herbs	Exotic	0.17
*Tamarindus indica *L.	Tamarindo	Tree	Exotic	0.17

Caprifoliaceae				
*Sambucus australis *Cham. & Schlecht	Flor de sabugo	Tree	Exotic	0.50

Clusiaceae				
*Symphonia *sp.	Bulandi	Herbs	—	0.17

Euphorbiaceae				
*Croton *sp.	Marmeleiro	Shrub	Native	0.17
*Euphorbia thymifolia*L.	Pé de pombo	Herbs	Native	0.67

Loranthaceae				
*Phthirusa pyrifolia *(H.B.K.) Eichl.	Esterco de passarinho	Herbs	Native	0.50

Malvaceae				
*Malva *sp.	Malva branca	Herbs	—	0.17

Poaceae				
*Brachiaria mutica *(Forsk.) Stapf	Capim de planta	Herbs	Exotic	0.33
*Dendrocalamus giganteus *Munro	Bambu	Tree	Exotic	0.17
*Saccharum officinarum *L.	Cana	Herbs	Exotic	0.17

Rhizophoraceae				
*Rhizophora mangle *L.	Mangue	Tree	Native	0.33

Rubiaceae				
*Cephaelis ipecacuanha *(Brot.) A. Rich.	Papeconha	Herbs	Native	0.50

Rutaceae				
*Pilocarpus *sp.	Jaborandi	—	—	0.33

Sapotaceae				
*Pradosia *sp.	Burinhê	—	—	0.33

Scrophulariaceae				
*Scoparia dulcis *L.	Vassourinha	Herbs	Exotic	0.17

RI: relative importance value.
